# AMPK-autophagy-mediated inhibition of microRNA-30a-5p alleviates morphine tolerance via SOCS3-dependent neuroinflammation suppression

**DOI:** 10.1186/s12974-022-02384-3

**Published:** 2022-01-29

**Authors:** Li Wan, Ru-Meng Jia, Lu-Lu Ji, Xin-Miao Qin, Liang Hu, Fan Hu, Yuan Han, Yin-Bing Pan, Chun-Yi Jiang, Wen-Tao Liu

**Affiliations:** 1 Jiangsu Key Laboratory of Neurodegeneration, Department of Pharmacology, Nanjing Medical University, Nanjing, 211166 Jiangsu China; 2 Department of Anesthesiology, Eye & ENT Hospital, Fudan University, Shanghai, 200031 China; 3 Department of Anesthesiology, The First Affiliated Hospital of Nanjing Medical University, Nanjing, 210029 Jiangsu China

**Keywords:** Morphine tolerance, AMPK-autophagy, microRNA-30a-5p, SOCS3, Neuroinflammation

## Abstract

**Background:**

The development of morphine tolerance is a clinical challenge for managing severe pain. Studies have shown that neuroinflammation is a critical aspect for the development of analgesic tolerance. We found that AMPK-autophagy activation could suppress neuroinflammation and improve morphine tolerance via the upregulation of suppressor of cytokine signaling 3 (SOCS3) by inhibiting the processing and maturation of microRNA-30a-5p.

**Methods:**

CD-1 mice were utilized for the tail-flick test to evaluate morphine tolerance. The microglial cell line BV-2 was utilized to investigate the mechanism of AMPK-autophagy-mediated posttranscriptional regulation of SOCS3. Proinflammatory cytokines were measured by western blotting and real-time PCR. The levels of SOCS3 and miRNA-processing enzymes were evaluated by western blotting, real-time PCR and immunofluorescence staining.

**Results:**

Based on experimental verification, miRNA-30a-5p could negatively regulate SOCS3. The AMPK activators AICAR, resveratrol and metformin downregulated miRNA-30a-5p. We found that AMPK activators specifically inhibited the processing and maturation of miRNA-30a-5p in microglia by degrading DICER and AGO2 via autophagy. Furthermore, a miRNA-30a-5p inhibitor significantly improved morphine tolerance via upregulation of SCOS3 in mice. It markedly increased the level of SOCS3 in the spinal cord of mice and subsequently inhibited morphine-induced phosphorylation of NF-κB p65. In addition, a miRNA-30a-5p inhibitor decreased the levels of IL-1β and TNF-α caused by morphine in microglia.

**Conclusion:**

AMPK-autophagy activation suppresses neuroinflammation and improves morphine tolerance via the upregulation of SOCS3 by inhibiting miRNA-30a-5p.

**Supplementary Information:**

The online version contains supplementary material available at 10.1186/s12974-022-02384-3.

## Background

The development of morphine tolerance significantly hinders the clinical utility of opioids necessitating repeated dose escalation regardless of disease progression. However, the underlying mechanism of antinociceptive tolerance induced by morphine remains elusive. The cellular and molecular mechanisms of morphine tolerance have been a focus of extensive research interest. In the last decade, an increasing number of studies have demonstrated that morphine can trigger neuroinflammation [1], which is characterized by the activation of microglia [2] and the increased production of proinflammatory cytokines, such as IL-1β, TNF-α and IL-6 [3–5], resulting in the enhancement of synaptic transmission and central sensitization [6]. In the neuroinflammation evoked by morphine, the mitogen-activated protein kinase (MAPK) family, including p38 MAPK and c-Jun N-terminal kinase (JNK), and the NF-κB signaling pathway were activated in microglia. TGF-β activated kinase 1 (TAK1), a member of the MAPK kinase kinase family, is the most common upstream kinase of MAPKs and plays an important role in regulating the TNF, IL-1 and TLR signaling pathways [7, 8]. Xu et al. reported that TAK1 activation was induced in association with the development of morphine tolerance [9]. In addition, numerous studies have reported that morphine causes the activation of microglia by binding with MD-2, a Toll-like receptor 4 (TLR4) accessory protein [10]. TLR4 is a pattern-recognition receptor that recognizes exogenous pathogen-associated molecular patterns (PAMPs, including lipopolysaccharide (LPS) and fungi) and endogenous danger-associated molecular patterns (DAMPs, including HMGB1 and HSP70) and subsequently initiates the immune response. It was reported that high mobility group box 1 (HMGB1) contributed to morphine tolerance via the activation of the TLR4/NF-κB pathway [11]. In addition, evidence has shown that morphine-induced HSP70 release activates microglia and triggers TLR4-mediated neuroinflammation by activating HSP70-TLR4-NLRP3 signaling [12]. Unfortunately, morphine tolerance is exceedingly difficult to manage by current drugs, and there is currently no quick and effective treatment. Accordingly, it is urgent to explore new mechanisms and develop treatment strategies.

Suppressor of cytokine signaling (SOCS) is a family of intracellular proteins with eight family members that negatively regulate inflammation [13]. Among the SOCS family members, SOCS3 is an important negative regulator in neuroinflammation [14]. Evidence has shown that SOCS3 inhibits IL-1 signaling by targeting the TRAF-6/TAK1 complex [15]. Upregulation of SOCS3 plays a vital role in pain relief. It has been reported that upregulating SOCS3 could attenuate postoperative pain via suppression of TLR4-mediated neuroinflammation [16]. Increasing studies of neuroinflammatory diseases such as multiple sclerosis [17] and experimental autoimmune encephalomyelitis [18] have shown that upregulation of SOCS3 could markedly inhibit the expression of proinflammatory cytokines. In our previous study [19], lidocaine inhibited TLR4-mediated neuroinflammation and alleviated morphine tolerance via adenosine monophosphate-activated protein kinase (AMP-activated protein kinase, AMPK)-mediated anti-inflammatory effects, and it was associated with the upregulation of SOCS3. Interestingly, the effect of AMPK activation on SOCS3 was at the protein level, as its mRNA remained unchanged. This implied that a posttranscriptional mechanism may be involved in this process. MicroRNAs (miRNAs) are short noncoding RNAs with a length of 21–23 nucleotides that are involved in the posttranscriptional regulation of gene expression by binding to specific mRNAs. A study showed that autophagy regulated miRNA activity by degrading DICER and AGO2. They are targeted for degradation as miRNA-free entities by the selective autophagy receptor NDP52 [20]. AMPK is a master sensor of the cellular energy status that is crucial for the adaptive response to limited energy availability [21]. Mammalian AMPK is known to be activated by falling cellular energy status, signaled by rising AMP/ATP and ADP/ATP ratios [22]. Under glucose starvation, AMPK could promote autophagy by directly activating ULK1 (unc-51-like autophagy-activating kinase 1) Ser317 and Ser777 [23]. In addition, it was reported that rapamycin, an autophagy activator, could attenuate morphine tolerance by suppressing the mTOR (mammalian target of rapamycin)–nNOS pathway [24]. At present, there is growing appreciation for the importance of activating AMPK in relieving morphine tolerance. Metformin, an activator of AMPK, could attenuate morphine tolerance by inhibiting morphine-induced activation of microglia [25]. However, the underlying mechanism is not fully understood. Hence, we investigated the relationship between AMPK activation, miRNA processing and SOCS3 regulation.

Based on bioinformatics prediction and experimental verification, we found that miRNA-30a-5p directly targets SOCS3. Here, we provide the first evidence that AMPK-autophagy activation inhibits miRNA-30a-5p by degrading DICER and AGO2, thereby inhibiting neuroinflammation to alleviate morphine tolerance.

## Methods

### Animals

Eight-week-old male CD-1 mice (18–22 g) were provided by the Experimental Animal Center at Nanjing Medical University, Nanjing, China. Animals were housed five to six per cage under pathogen-free conditions with soft bedding under controlled temperature (22 ± 2 °C) and a 12-h light/dark cycle (lights on at 8:00 a.m.). Behavioral testing was performed during the light cycle (between 9:00 a.m. and 5:00 p.m.). We habituated animals to the testing environments for 2 days before starting experiments. For each group of experiments, the animals were matched by age and body weight.

### Chemicals and reagents

Metformin hydrochloride was purchased from MedChem Express (USA). Morphine hydrochloride was purchased from Shenyang First Pharmaceutical Factory, Northeast Pharmaceutical Group Company (China). Rapamycin and bafilomycin were purchased from MedChem Express (USA). SOCS3 siRNA, Atg5 siRNA, miRNA-30a-5p inhibitor, miRNA-30a-5p mimic, miRNA-203-3p mimic, miRNA-19a-3p mimic, miRNA-455-5p mimic, miRNA-218-5p mimic, control siRNA, control microRNA mimic and control microRNA inhibitor were purchased from GenePharma (China). In vivo-jetPEI^®^ was purchased from Polyplus-transfection. Antibodies against β-actin, SOCS3, phosphorylated p38 (Tyr182), phosphorylated NF-κB p65 (Ser536), NF-κB p65, AGO1, AGO2 and DICER were purchased from ABclonal (China). Antibodies against p38, phosphorylated AMPK (Thr172), Atg5 and LC3 were purchased from Cell Signaling Technology (USA). Secondary antibodies were from Sigma-Aldrich (USA). Immunofluorescent antibody for IBA-1 was purchased from Abcam (USA). Immunofluorescent antibody for SOCS3 was purchased from ABmart (China). Immunofluorescent antibody for GFAP was purchased from Santa Cruz Biotechnology (USA). Immunofluorescent antibody for NeuN was purchased from Millipore (USA). Secondary antibodies for immunofluorescence were as follows: Alexa Fluor 488-conjugated donkey anti-rabbit and Alexa Fluor 647-conjugated donkey anti-goat were purchased from Invitrogen. TaqMan™ Universal Master Mix II, no UNG, TaqMan™ MicroRNA Reverse transcription kit, TaqMan^®^ MicroRNA assays and TaqMan^®^ pri-MiRNA assays were purchased from Thermo Fisher Scientific (USA). Fetal bovine serum (FBS) was purchased from Biological Industries, and other cell culture media and supplements were purchased from KenGEN (China). HiScript^®^ RT SuperMix and SYBR Green were purchased from Vazyme (China).

### Behavioral testing

Behavioral testing was carried out in a blinded manner. A hot water tail-flick test was performed to measure the analgesic effect. Behavioral testing was performed 30 min after morphine administration by tail-flick assay every morning. Mice were gently held in a tender towel. One-third of the tail was immersed in 52 ± 0.5 °C hot water. The latency until tail withdrawal from the bath was determined. The cutoff time was 10 s to avoid tissue damage. Morphine was administered intrathecally at 10 μg for 7 consecutive days to establish chronic antinociceptive tolerance. Data were calculated as a percentage of maximal possible effect (%MPE), which was calculated by the following formula: 100% × [(Drug response time − Basal response time)/(10 s − Basal response time)] = %MPE.

### Intrathecal injection procedure

To perform intrathecal (i.t.) injections, the mice were placed in a prone position, and the midpoint between the tips of the iliac crest was located. A Hamilton syringe with a 30-gauge needle was inserted into the subarachnoid space of the spinal cord between the L4 and L5 spinous processes. Proper intrathecal injection was systemically confirmed by observation of a tail flick. Intrathecal injection did not affect baseline responses compared with latencies recorded before injection.

### Cell culture

BV-2 (microglia) and c8-DA cells (astrocytes) were maintained in humidified 5% CO_2_ at 37 °C in Dulbecco’s modified Eagle’s medium (DMEM; KenGEN BioTECH, China) supplemented with 10% (v/v) FBS (Biological Industries), 80 U/mL penicillin, and 0.08 mg/mL streptomycin. SH-SY5Y cells (neurons) were maintained in humidified 5% CO_2_ at 37 °C in Modified Eagle Media: F-12 (MEM/F12, Gibco, NY, United States) supplemented with 10% (v/v) FBS (Biological Industries), 80 U/mL penicillin and 0.08 mg/mL streptomycin. For further experiments, 10^5^ cells were plated in a 6-well plate overnight. Cell extracts were analyzed by immunoblot assay or real-time PCR.

### Western blot

Samples (cells or spinal cord tissue segments at L4–L5) were collected and washed with ice-cold PBS before being lysed in radioimmunoprecipitation assay (RIPA) lysis buffer, and then sample lysates were separated by SDS-PAGE and electrophoretically transferred onto polyvinylidene fluoride membranes (Millipore). The membranes were blocked with 10% low-fat dry powdered milk or with 5% BSA and 5% low-fat dry powdered milk in TBST (Tris–HCl, NaCl, Tween 20) for 2 h at room temperature and then probed with primary antibodies at 4 °C overnight. Finally, horseradish peroxidase (HRP)-coupled secondary antibodies (Sigma, USA) were utilized to detect the corresponding primary antibody. The dilution factors of the primary antibodies were β-actin (1:10,000), p38 (1:1000), p-p38 (Tyr182) (1:1000), p65 (1:1000), p-p65 (Ser536) (1:1000), p-AMPK (Thr172) (1:1000), AGO1 (1:300), AGO2 (1:1000), DICER (1:800), SOCS3 (1:1000), Atg5 (1:1000) and LC3 (1:1000). The bands were developed by enhanced chemiluminescence reagents (New Cell & Molecular Biotech Co., Ltd, China). Data were collected with the Molecular Imager and analyzed with ImageJ software (NIH, United States).

### Immunohistochemistry

Under deep anesthesia by intraperitoneal injection of pentobarbital sodium (50 mg/kg), animals were perfused with normal saline followed by 4% paraformaldehyde in 0.1 M PBS, pH 7.2–7.4, for 20 min. Then, the L4 and L5 lumbar segments were dissected out and postfixed in the same fixative. The embedded blocks were sectioned at a thickness of 15 μm and processed for immunofluorescence assays. Sections from each group (three mice in each group) were incubated with primary antibodies against IBA-1 (1:500), SOCS3 (1:50), GFAP (1:300), and NeuN (1:300). Then, the freefloating sections were washed with PBS and incubated with the secondary antibody (1:1000) for 2 h at room temperature. After being washed three times with PBS for 10 min each time, the samples were investigated with a confocal microscope (Zeiss LSM710, Germany).

The average fluorescence intensity of microglia in dorsal horn lamina I–III of the spinal cord (labeled with white dotted line) was analyzed by ImageJ software and normalized to the saline-treated group (*n* = 3). The percentage of SOCS3-immunopositive cells in microglia, neurons and astrocytes in dorsal horn lamina I–III of the spinal cord (labeled with white dotted line) was analyzed by ImageJ software. Data were collected from three random areas of lamina I–III from each dorsal horn section (*n* = 3).

### Transfection

SOCS3 siRNA, Atg5 siRNA, miRNA-30a-5p inhibitor, miRNA-30a-5p mimic, miRNA-203-3p mimic, miRNA-19a-3p mimic, miRNA-455-5p mimic, miRNA-218-5p mimic, control siRNA, control microRNA mimic and control microRNA inhibitor were purchased from GenePharma (China). Control siRNA, control microRNA mimic and control microRNA inhibitor were used as negative controls. For the transfection of siRNA, microRNA mimics and microRNA inhibitor in vitro, cells were cultured in 6-well plates with antibiotic-free medium the day before transfection. Transfection was conducted when cells reached 50–70% confluence using Lipofectamine 2000 (Invitrogen, USA) and serum-free medium according to the manufacturer’s instructions. After 4 h, the transfection medium was replaced with culture medium containing 10% FBS and then incubated at 37 ℃ in 5% CO_2_. For the in vivo study, mice were intrathecally injected with microRNA-30a-5p inhibitor complexed with transfection reagent in vivo*-*jetPEI (Polyplus-transfection, France). The sequences of siRNA, microRNA mimics and microRNA inhibitor used in this study are listed in Table 1.

**Table 1:** Sequences for transfection

Name	Sequence (5ʹ–3ʹ)
*Socs3* siRNA (mouse)	
Sense	GUAUGAUGCUCCACUUUAATT
Anti-sense	UUAAAGUGGAGCAUCAUACTT
*Atg5* siRNA (mouse)	
Sense	CAUCAACCGGAAACUCAUTT
Anti-sense	AUGAGUUUCCGGUUGAUGTT
microRNA-30a-5p mimic	
Sense	UGUAAACAUCCUCGACUGGAAG
Anti-sense	UCCAGUCGAGGAUGUUUACAUU
microRNA-203-3p mimic	
Sense	GUGAAAUGUUUAGGACCACUAG
Anti-sense	AGUGGUCCUAAACAUUUCACUU
microRNA-19a-3p mimic	
Sense	UGUGCAAAUCUAUGCAAAACUGA
Anti-sense	AGUUUUGCAUAGAUUUGCACAUU
microRNA-455-5p mimic	
Sense	UAUGUGCCUUUGGACUACAUCG
Anti-sense	AUGUAGUCCAAAGGCACAUAUU
microRNA-218-5p mimic	
Sense	UUGUGCUUGAUCUAACCAUGU
Anti-sense	AUGGUUAGAUCAAGCACAAUU
microRNA-30a-5p inhibitor	CUUCCAGUCGAGGAUGUUUACA
Control siRNA	
Sense	UUCUCCGAACGUGUCACGUTT
Anti-sense	ACGUGACACGUUCGGAGAATT
Control mimic	
Sense	UUCUCCGAACGUGUCACGUTT
Anti-sense	ACGUGACACGUUCGGAGAATT
Control microRNA inhibitor	CAGUACUUUUGUGUAGUACAA

### Quantitative real-time PCR

Total RNA was extracted from BV-2 cells (microglia), c8-DA cells (astrocytes) and SH-SY5Y cells (neurons) using TRIzol reagent (Invitrogen, USA). (1) Isolated RNA was reverse transcribed into cDNA using HiScript^®^ RT SuperMix (Vazyme, China) following standard protocols. Quantitative real-time PCR (qRT-PCR) was performed with synthetic primers and SYBR Green (Vazyme, China) with a QuantStudio 5 Real-Time PCR Detection System (Thermo Fisher Scientific, USA). The relative expression levels of *Socs3*, *Il1b* and *Tnfa* were calculated and quantified with the 2^−ΔΔCt^ method after normalization to the reference β-actin expression. (2) Isolated RNA was reverse transcribed into cDNA using TaqMan^RT^ MicroRNA (Thermo Fisher Scientific, USA). MiRNA-30a-5p was measured using TaqMan™ MicroRNA Assays (Thermo Fisher Scientific, USA) in accordance with the manufacturer’s instructions. The relative expression level of miRNA-30a-5p was calculated and quantified with the 2^−ΔΔCt^ method after normalization to the reference U6 expression. (3) Isolated RNA was reverse transcribed into cDNA using HiScript^®^ RT SuperMix (Vazyme, China) following standard protocols. *MiRNA-30a* (pri-miRNA-30a-5p) was measured using TaqMan™ Pri-miRNA Assays (Thermo Fisher Scientific, USA) in accordance with the manufacturer’s instructions. The relative expression level of *miRNA-30a* was calculated and quantified with the 2^−ΔΔCt^ method after normalization to the reference β-actin expression. The primers used for quantitative real-time PCR in this study are listed in Table 2.

**Table 2:** Sequences of primers for real-time quantitative polymerase chain reaction

Gene	Sequence (5ʹ–3ʹ)
*β-actin (mouse)*	
Forward	CATTGCTGACAGGATGCAGAAGG
Reverse	TGCTGGAAGGTGGACAGTGAGG
*Socs3 (mouse)*	
Forward	GCTCCAAAAGCGAGTACCAGC
Reverse	AGTAGAATCCGCTCTCCTGCAG
*Tnfα (mouse)*	
Forward	CATCTTCTCAAAATTCGAGTGA
Reverse	TGGGAGTAGACAAGGTACAA
*Il1b (mouse)*	
Forward	TCATTGTGGCTGTGGAGAAG
Reverse	AGGCCACAGGTATTTTGTCG

*Il-1b* interleukin-1β, *Tnf-a* tumor necrosis factor-α, *Socs3* suppressor of cytokine signaling 3

### Statistical analysis

GraphPad Prism 9 software (GraphPad Software, San Diego, CA, USA) was used to conduct all statistical analyses. The differences between two groups were evaluated by Student’s *t* test. The data from more than two groups were evaluated by one-way ANOVA or two-way ANOVA. The results are presented as the mean ± SEM of independent experiments. The results described as significant were based on a criterion of *p* < 0.05.

## Results

### Metformin suppressed neuroinflammation and improved morphine tolerance via upregulation of SOCS3 in mice

**Fig. 1:**
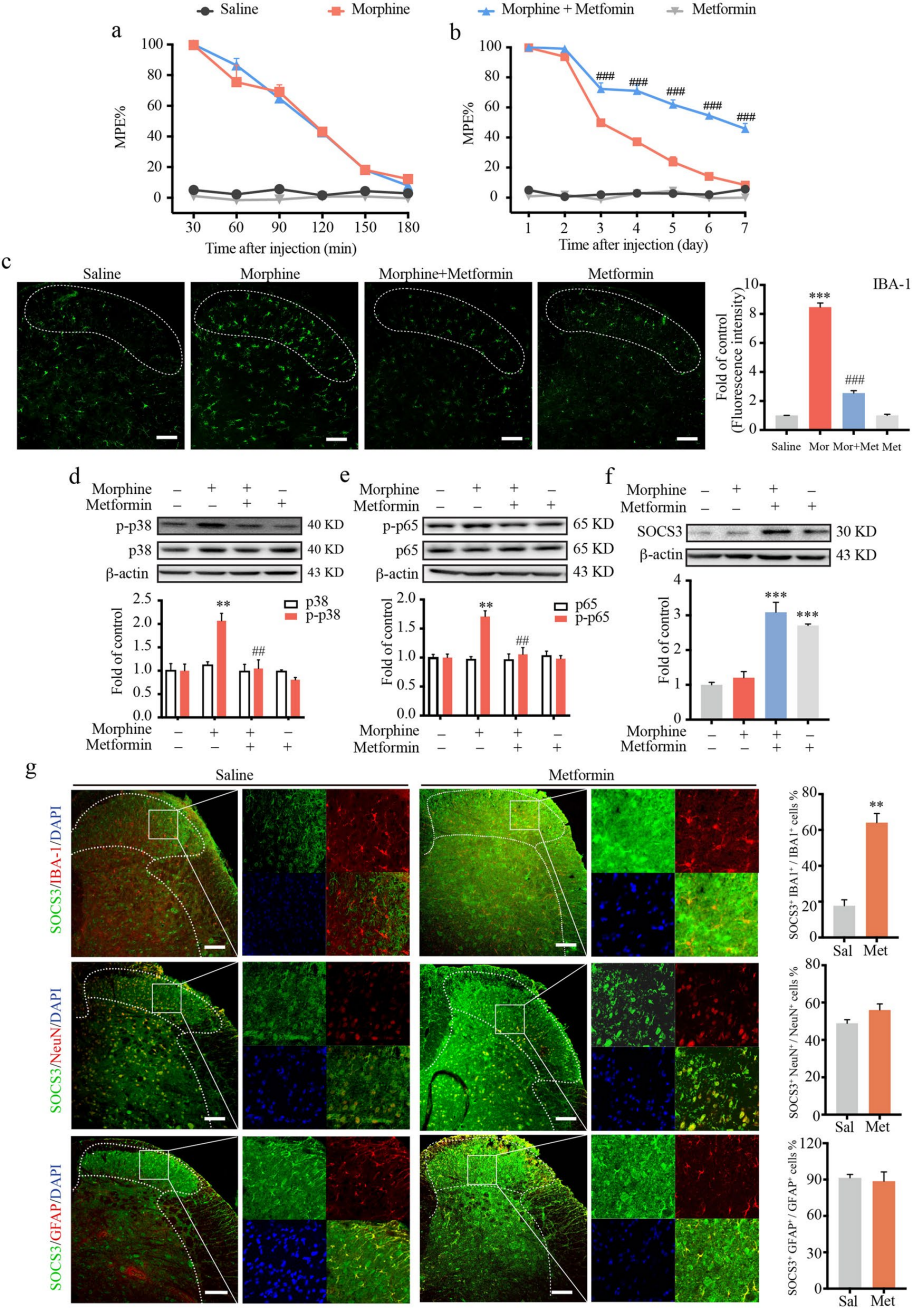
Metformin suppressed morphine-induced microglial activation and attenuated morphine tolerance. The tail-flick test was performed to evaluate the effect of metformin on morphine tolerance. Data are shown as a percentage of maximal possible effect (%MPE). **a** Metformin had no effect on acute morphine analgesic effect. Mice were pretreated with metformin (200 mg/kg, i.g.) 12 h before morphine (10 μg/μL, i.t.) administration. MPE was measured every 30 min after morphine injection (*n* = 8). **b** Metformin suppressed chronic morphine tolerance. Mice were injected intrathecally with morphine (10 μg/μL, i.t.) once daily for 7 days, and the MPE was measured 30 min after the injection of each day. Mice were pretreated with metformin (200 mg/kg, i.g.) 12 h before morphine administration everyday. **c** Representative immunofluorescence images showed the effect of metformin on the morphine-induced activation of microglia in dorsal horn lamina I–III of the spinal cord (labeled with a white dotted line) (*n* = 3). **d**, **e** The phosphorylation of p38 and p65 in the spinal cord was evaluated at day 7 by western blot (*n* = 3). **f** The protein level of SOCS3 in the spinal cord was evaluated at day 7 by western blot (*n* = 3). **g** Mice were treated with metformin (200 mg/kg, i.g.) once daily for 7 days. Spinal cord samples were collected after the last administration of metformin. Representative confocal microscopy study of the expression of SOCS3 (green) in microglia (IBA-1, red), neurons (NeuN, red) and astrocytes (GFAP, red) of dorsal horn lamina I-III of the spinal cord (labeled with white dotted line) (*n* = 3). Data were analyzed by two-way ANOVA in **a** and **b**. Data were analyzed by one-way ANOVA in **c**–**f**. Data were analyzed by Student’s *t* test in **g**. **p < .01, ***p < .001 vs. saline; ^##^*p* < 0.01, ^###^*p* < .001 vs. morphine-treated group. Scale bars = 100 μm

It is widely accepted that morphine can induce neuroinflammation, which is characterized by the activation of microglia and neuroinflammation. To investigate the effects of metformin on morphine-induced neuroinflammation in vivo, mice were administered metformin (200 mg/kg, i.g.) 12 h before morphine injection (10 μg/10 μL, i.t.) once daily for 7 days. We measured the latency of tail withdrawal in mice by tail-flick assay. Metformin significantly improved chronic morphine tolerance (Fig. 1b) and did not change the acute analgesic effect (Fig. 1a). Our immunofluorescence staining data showed that repeated morphine treatment once daily for 7 days (10 μg/10 μL, i.t.) resulted in the activation of microglia in spinal cord lamina I–III (IBA-1 as a microglial marker), and metformin (200 mg/kg, i.g.) nearly completely inhibited the activation of microglia (Fig. 1c). Furthermore, to determine the anti-inflammatory effect of metformin, we examined the phosphorylation of p38 mitogen-activated protein kinase (MAPK) and NF-κB p65 in the spinal cord of mice. As indicated in Fig. 1d, e, immunoblot data demonstrated that metformin remarkably suppressed morphine-induced phosphorylation of p38 and p65.

To investigate the mechanism underlying the anti-neuroinflammatory effects of metformin, we investigated suppressor of cytokine signaling (SOCS) 3, an endogenous immune brake that inhibits neuroinflammation. As shown in Fig. 1f, the protein level of SOCS3 was increased after metformin administration.

Moreover, to analyze the expression of SOCS3 after metformin treatment (200 mg/kg, i.g.), confocal microscopic scanning in the spinal cord was performed. Metformin increased SOCS3 colocalization with IBA-1 (a microglial marker) from 17.7 to 64.0% in spinal cord lamina I–III and did not obviously change SOCS3 colocalization with NeuN (a neuronal marker) or GFAP (an astrocyte marker) (Fig. 1g). To further confirm the above data, we cultured a neuronal cell line (SH-SY5Y cells) and astrocyte cell line (C8-DA cells). SH-SY5Y cells and C8-DA cells were treated with metformin (2.5 mM, 12 h). Immunoblot data showed that metformin did not affect the expression of SOCS3 in either SH-SY5Y cells or C8-DA cells (Additional file 1: Fig. S1a and b).

### Metformin significantly suppressed the morphine-induced inflammatory response in an SOCS3-dependent manner in a microglial cell line

**Fig. 2:**
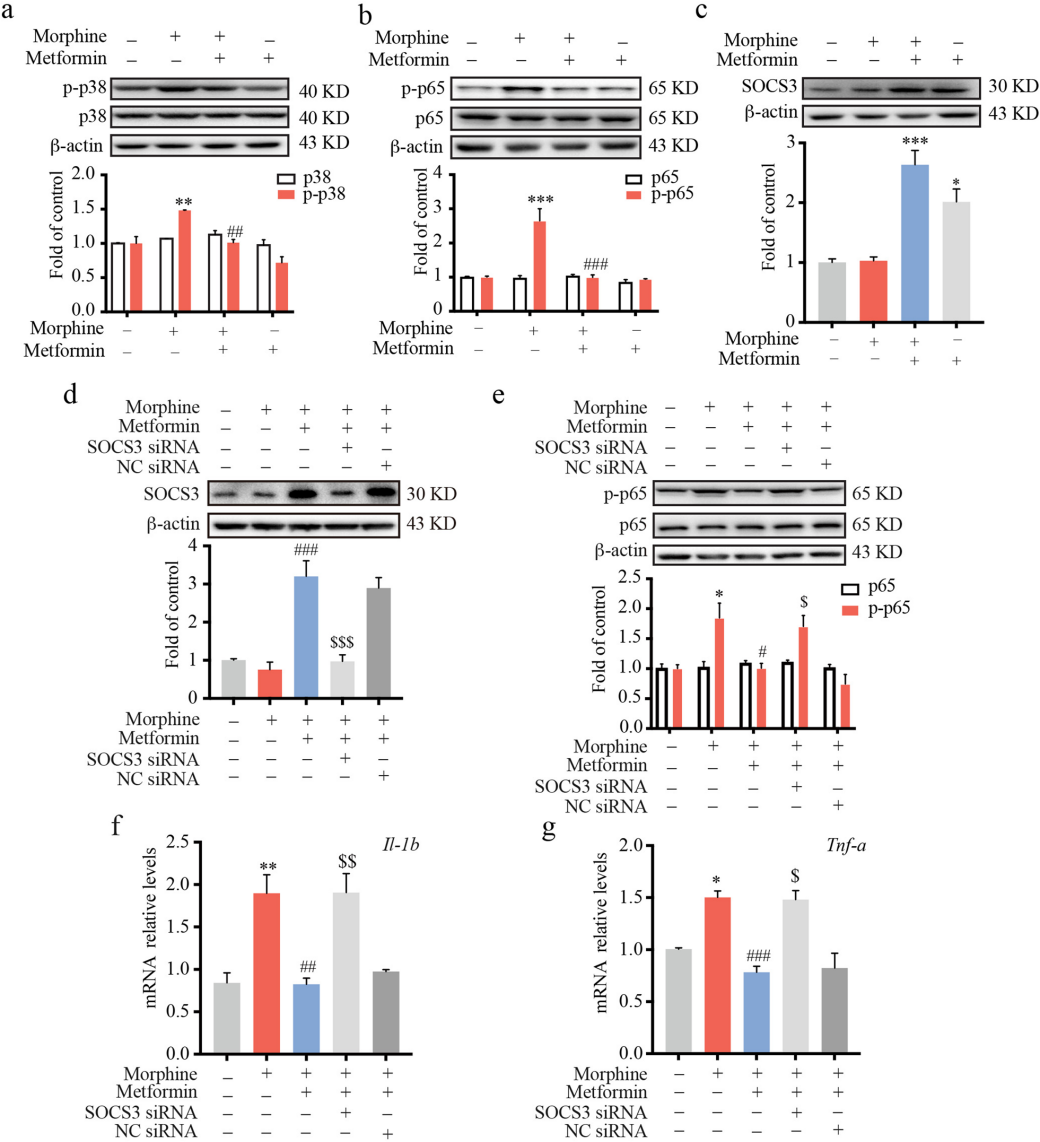
Metformin significantly suppressed the morphine-induced inflammatory response in a SOCS3-dependent manner in BV-2 cells. BV-2 cells were pretreated with metformin (2.5 mM) for 12 h and then exposed to morphine (200 μM) for 12 h. Cells were collected and analyzed. **a**–**c** Western blot analysis showed the phosphorylation of p38 (**a**) and p65 (**b**) and the protein level of SOCS3 (**c**) (*n* = 3). BV-2 cells were transfected with 100 pmol SOCS3 siRNA or negative control (NC) siRNA for 36 h. Then, the cells were subjected to metformin (2.5 mM) for 12 h, followed by exposure to morphine (200 μM) for 12 h. Cell extracts were collected and analyzed. **d**, **e** The protein level of SOCS3 (**d**) and phosphorylation of p65 (**e**) were evaluated by western blot (*n* = 3). **f**, **g** The mRNA levels of *Il1b* (**f**) and *Tnfa* (**g**) were evaluated by real-time PCR (*n* = 3). Data were analyzed by one-way ANOVA. *p < .05, **p < .01, ***p < .001 vs. the control group; ^#^*p* < .05, ^##^*p* < .01, ^###^*p* < .001 vs. the morphine-treated group. ^$^*p* < .05, ^$$^*p* < .01, ^$$$^*p* < .001 vs. morphine and metformin-coadministered group

Accumulating evidence indicates that neuroinflammation contributes to central sensitization after chronic morphine treatment through microglial activation. To investigate the effects of metformin on morphine-induced microglial activation in vitro, immortalized murine microglial BV-2 cells were utilized. BV-2 cells were pretreated with metformin (2.5 mM, 12 h) before morphine (200 μM, 12 h) stimulation. As shown in Fig. 2a, b, morphine administration largely increased the phosphorylation of p38 and p65. Metformin remarkably inhibited the phosphorylation of p38 and p65 induced by morphine. To test the effect of metformin on SOCS3 in BV-2 cells, immunoblotting was performed. Consistent with the in vivo* results*, immunoblot data showed that metformin could increase the expression of SOCS3 (Fig. 2c).

To investigate whether the anti-inflammatory effects of metformin were SOCS3-dependent, SOCS3 small interfering RNA (siRNA) was utilized to downregulate SOCS3. As shown in Fig. 2d, SOCS3 siRNA abolished the effect of metformin on the upregulation of SOCS3. Western blot data showed that SOCS3 siRNA also abrogated the inhibitory effect of metformin on the activation of NF-κB induced by morphine (Fig. 2e). In accordance with the above, real-time PCR data demonstrated that the knockdown of SOCS3 suppressed the effects of metformin on the mRNA levels of *Il-1b* and *Tnf-a* in morphine-stimulated BV-2 cells (Fig. 2f, g).

### AMPK activators decreased the level of microRNA-30a-5p, which directly targeted SOCS3

**Fig. 3:**
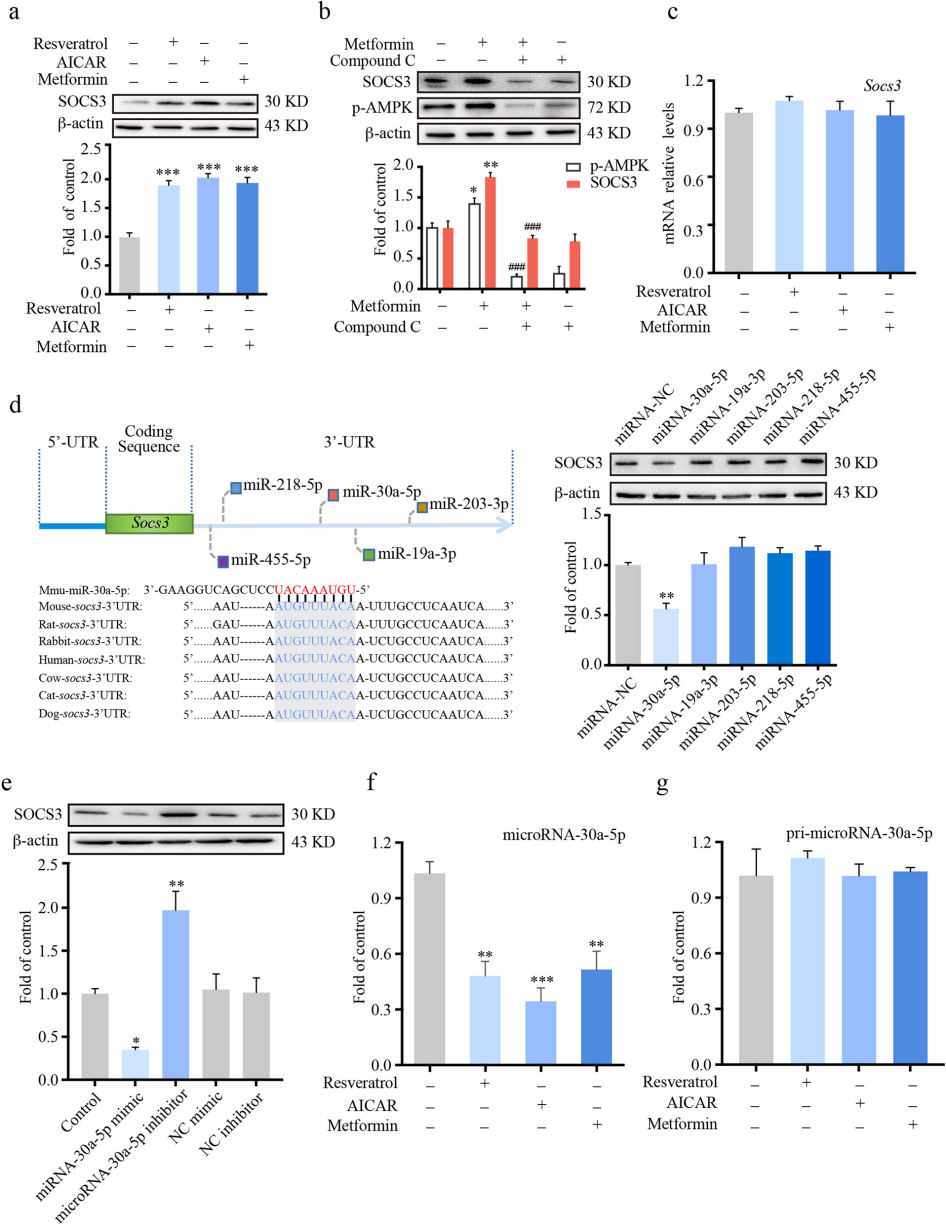
AMPK activators upregulated the protein level of SOCS3 via the inhibition of microRNA-30a-5p. **a** BV-2 cells were treated with resveratrol (50 μM), AICAR (300 μM) or metformin (2.5 mM) for 12 h. Cell extracts were collected. The protein level of SOCS3 was evaluated by western blot (*n* = 3). **b** BV-2 cells were subjected to compound C (AMPK inhibitor, 20 μM) for 12 h, followed by exposure to metformin (2.5 mM) for 12 h. The protein level of SOCS3 and phosphorylation of AMPK were tested by western blot (*n* = 3). **c** BV-2 cells were treated with resveratrol (50 μM), AICAR (300 μM) or metformin (2.5 mM) for 12 h. The mRNA level of *Socs3* was evaluated by real-time PCR (*n* = 3). **d** Screening based on bioinformatics (http://www.targetscan.org), five miRNAs were candidates that may target SOCS3. BV-2 cells were transfected with 100 pmol miRNA-30a-5p mimic, miRNA-19a-3p mimic, miRNA-203-5p mimic, miRNA-218-5p mimic, miRNA-455-5p mimic or negative control (NC) miRNA for 36 h. The protein level of SOCS3 was evaluated by western blot (*n* = 3). **e** BV-2 cells were transfected with 100 pmol miRNA-30a-5p mimic, miRNA-30a-5p inhibitor, negative control mimic or negative control inhibitor for 36 h. Western blot analysis showed the protein level of SOCS3 (*n* = 3). BV-2 cells were treated with resveratrol (50 μM), AICAR (300 μM) or metformin (2.5 mM) for 12 h. **f** The level of miRNA-30a-5p was evaluated by real-time PCR (*n* = 3). **g** The level of *miRNA-30a* (primary miRNA-30a-5p) was evaluated by real-time PCR (*n* = 3). Data were analyzed by one-way ANOVA. *p < .05, **p < .01, ***p < .001 vs. control group. ^##^*p* < .01, ^###^*p* < .001 vs. metformin-treated group

Metformin, a biguanide class of antidiabetic drugs and a classic activator of AMPK, has a remarkable anti-inflammatory effect. Our previous study showed that lidocaine alleviated morphine tolerance via AMPK-mediated upregulation of SOCS3. However, the activation of AMPK did not elevate the transcriptional level of SOCS3. This implied that a posttranscriptional mechanism may be involved. Therefore, we tested the effects of resveratrol, AIACR and metformin as AMPK activators on the regulation of SOCS3. BV-2 cells were treated with resveratrol (50 μM, 12 h), AICAR (300 μM, 12 h) or metformin (2.5 mM, 12 h), and then immunoblot data showed that AMPK activators increased the protein level of SOCS3 (Fig. 3a). Compound C, an AMPK inhibitor, abolished metformin-induced upregulation of SOCS3 (Fig. 3b). Interestingly, resveratrol, AIACR and metformin did not change the level of *Socs3* mRNA (Fig. 3c). Therefore, we hypothesized that AMPK activation probably affected SOCS3 via miRNAs.

MiRNAs comprise a large family of ~ 21-nucleotide-long RNAs that have emerged as key posttranscriptional regulators of gene expression. We screened the database based on bioinformatics predictions and found five miRNA mimics, miRNA-30a-5p, miRNA-203-3p, miRNA-19a-3p, miRNA-455-5p and miRNA-218-5p, which were conserved in targeting SOCS3 among different species (Fig. 3d). By experimental verification, we found that the miRNA-30a-5p mimic could negatively regulate SOCS3 (Fig. 3d). Furthermore, as shown in Fig. 3e, the miRNA-30a-5p inhibitor abrogated the effect of the miRNA-30a-5p mimic on SOCS3. To investigate the effect of AMPK activation on miRNA-30a-5p, real-time PCR was performed. The results showed that the administration of resveratrol, AICAR or metformin to BV-2 cells decreased the level of miRNA-30a-5p (Fig. 3f) but had no effect on the level of pri-miRNA-30a-5p (primary microRNA-30a-5p) (Fig. 3g). It was obvious that miRNA-30a-5p directly targeted SOCS3 and was downregulated by AMPK activators.

### MicroRNA-30a-5p inhibitor improved morphine tolerance via neuroinflammation suppression in mice

**Fig. 4:**
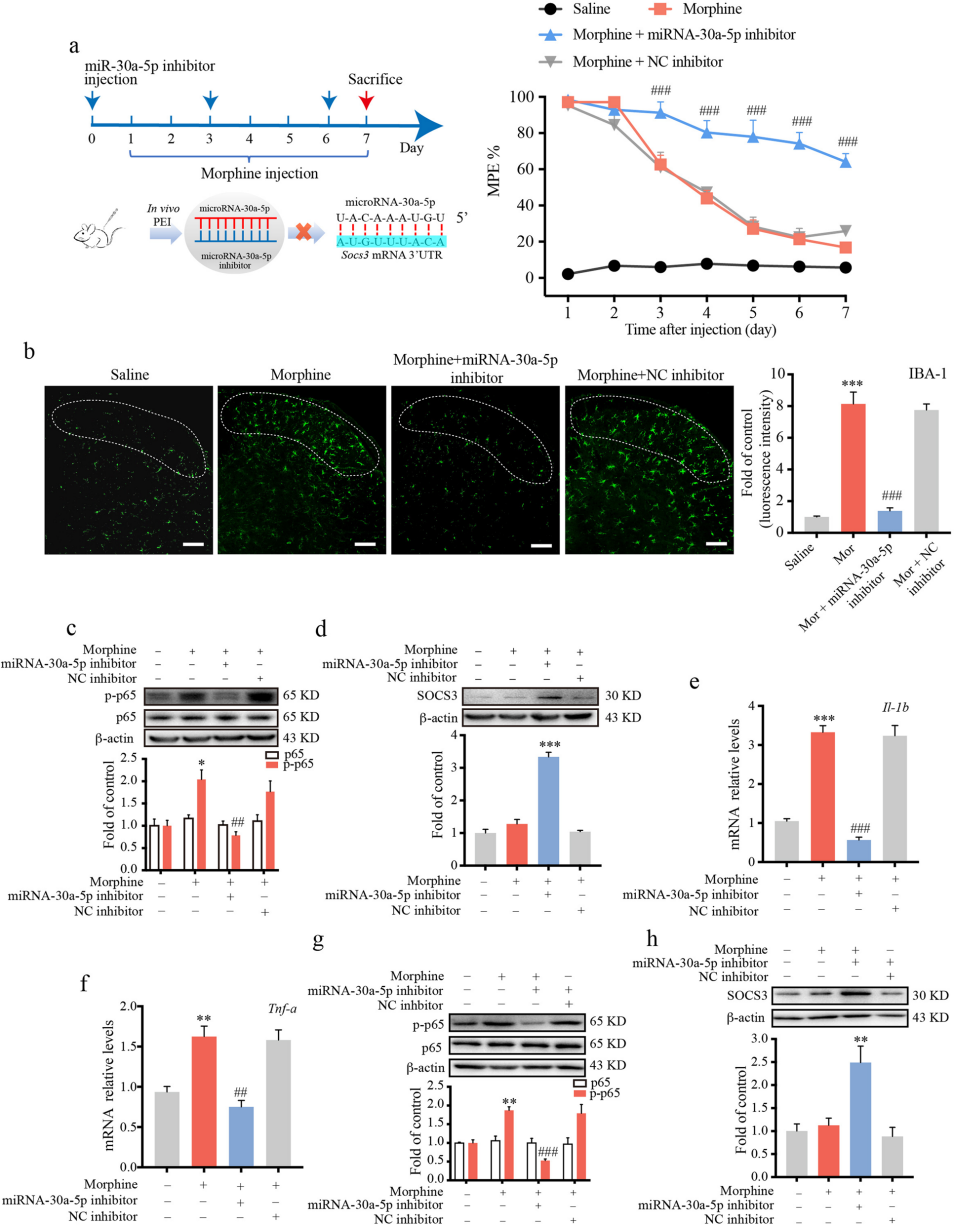
MicroRNA-30a-5p inhibitor improved morphine tolerance by inhibiting microglial activation in mice. Mice were intrathecally injected with morphine (10 μg) each day for 7 days and miRNA-30a-5p inhibitor (125 pmol) with in vivo jetPEI^®^ 1 day before morphine injection (10 μg) and on day 3 and 6. The tail-flick test was performed to evaluate the effect of the miRNA-30a-5p inhibitor on morphine tolerance. Data are shown as a percentage of maximal possible effect (%MPE). **a** The tail-flick test was performed 30 min after morphine injection each day (*n* = 8). **b** Representative immunofluorescence images showing the effect of miRNA-30a-5p inhibitor on the activation of microglia (IBA-1, green) evoked by morphine in the dorsal horn lamina I–III of spinal cord (labeled with white dotted line) (*n* = 3). **c** The phosphorylation of p65 was evaluated by western blot in the spinal cord (*n* = 3). **d** Western blot analysis showed the protein level of SOCS3 in the spinal cord (*n* = 3). BV-2 cells were transfected with 100 pmol miRNA-30a-5p inhibitor or negative control (NC) inhibitor for 36 h, followed by morphine (200 μM) treatment for 12 h. **e** The mRNA level of *Il1b* was tested by real-time PCR. **f** The mRNA level of *Tnfa* was evaluated by real-time PCR (*n* = 3). **g** Representative western blot data showed the phosphorylation of p65 (*n* = 3). **h** The protein level of SOCS3 was tested by western blot (*n* = 3). Data in **a** was analyzed by two-way ANOVA. Data in **b**–**h** were analyzed by one-way ANOVA. *p < .05, **p < .01, ***p < .001 vs. saline; ^##^*p* < .01, ^###^*p* < .001 vs. morphine-treated group. Scale bar = 100 μm

To explore the role of miRNA-30a-5p inhibitor in the modulation of morphine tolerance, we examined whether spinal miRNA-30a-5p inhibition could affect morphine-evoked neuroinflammation. We designed and synthesized a miRNA-30a-5p inhibitor with 2ʹ-*O*-methylation (2ʹ-O-Me) modification. Mice were intrathecally injected with miRNA-30a-5p inhibitor (125 pmol/10 μL) 1 day before morphine injection (10 μg/10 μL, once daily for 7 days, i.t.) and on day 3 and 6 to inhibit the function of miRNA-30a-5p (Fig. 4a). Our behavioral observations suggested that the miRNA-30a-5p inhibitor remarkably improved morphine tolerance. In addition, our immunofluorescence staining data showed that repeated morphine treatment induced the activation of microglia (IBA-1 as a microglial marker), and the miRNA-30a-5p inhibitor significantly inhibited the activation of microglia in spinal cord lamina I–III (Fig. 4b). Consistent with the behavioral data, the immunoblot results demonstrated that the miRNA-30a-5p inhibitor suppressed the morphine-induced phosphorylation of NF-κB p65 (Fig. 4c) in the spinal cord of mice via the upregulation of SOCS3 (Fig. 4d).

In addition, BV-2 cells were also transfected with 100 pmol miRNA-30a-5p inhibitor for 36 h followed by morphine (200 μM, 12 h) stimulation. Immunoblot results showed that the suppressive effect of the miRNA-30a-5p inhibitor on morphine-induced activation of NF-κB p65 (Fig. 4g) via the upregulation of SOCS3 (Fig. 4h). Real-time PCR demonstrated that the miRNA-30a-5p inhibitor suppressed *Il-1b* mRNA (Fig. 4e) and *Tnf-a* mRNA (Fig. 4f) in morphine-stimulated BV-2 cells. Our results fully clarified that miRNA-30a-5p is a key target to regulate SOCS3 in improving morphine tolerance.

### AMPK activators upregulated SOCS3 by enhancing autophagy to suppress microRNA-30a-5p

**Fig. 5:**
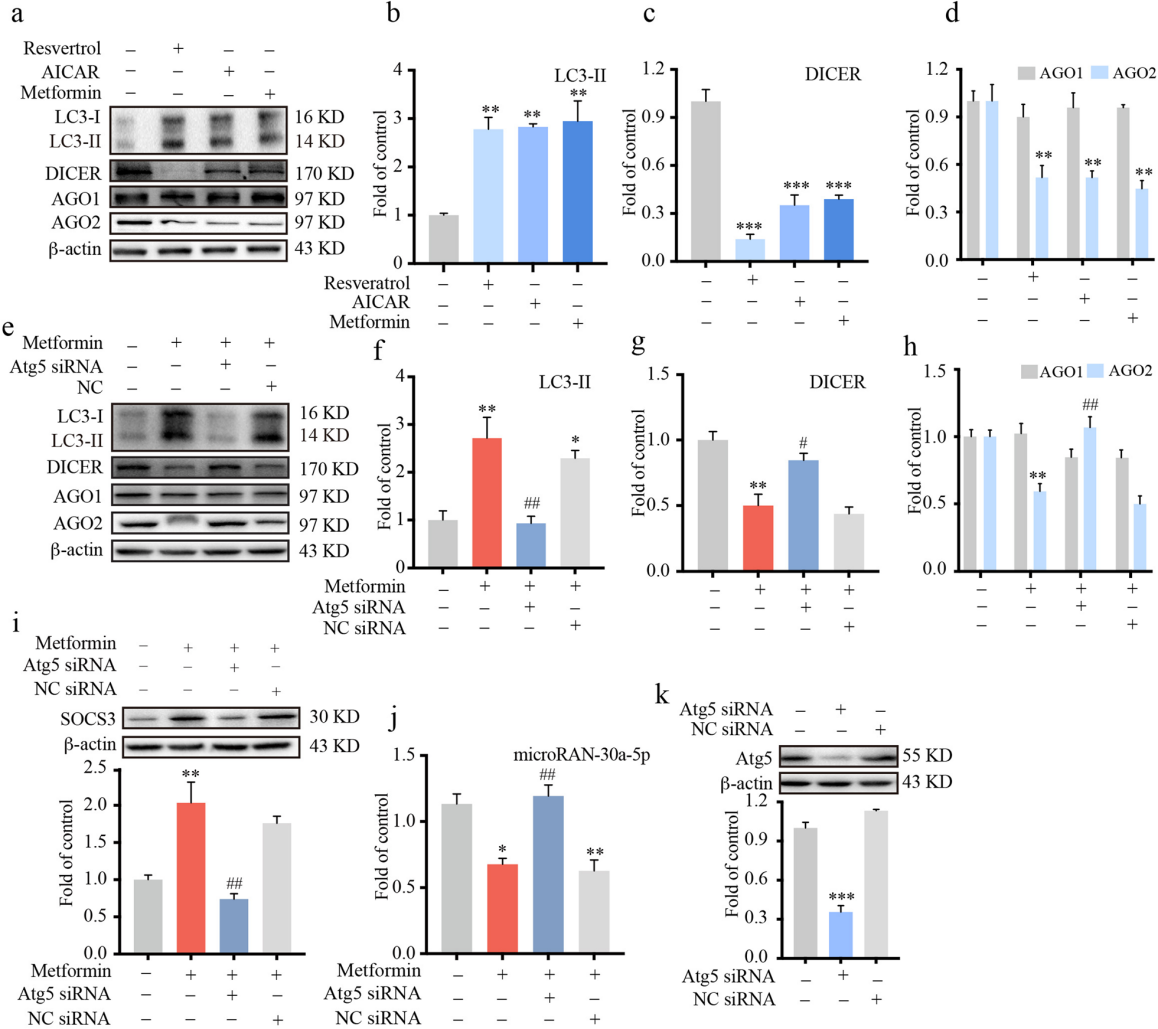
AMPK activators upregulated SOCS3 by enhancing autophagy to suppress microRNA-30a-5p. **a**–**d** BV-2 cells were treated with resveratrol (50 μM), AICAR (300 μM) or metformin (2.5 mM) for 12 h. Cell extracts were collected, and the protein levels of LC3-II (**b**), DICER (**c**), AGO1 and AGO2 (**d**) were evaluated by western blotting (*n* = 3). BV-2 cells were transfected with 100 pmol Atg5 siRNA or negative control (NC) siRNA for 36 h and then subjected to metformin (2.5 mM) for 12 h. The efficiency of Atg5 knockdown was assessed by immunoblot assay (**k**) (*n* = 3). The protein levels of LC3-II, DICER, AGO1, AGO2 and SOCS3 were evaluated by western blot (**e**–**i**) (*n* = 3). **j** The level of miRNA-30a-5p was evaluated by real-time PCR after metformin administration (*n* = 3). Data were analyzed by one-way ANOVA. *p < .05, **p < .01, ***p < .001 vs. the control group; ^#^*p* < .05, ^##^*p* < .01 vs. the metformin-treated group

Next, we explored the mechanisms underlying the downregulation of miRNA-30a-5p by AMPK activation. It was reported that autophagy is required for homeostasis and activity of miRNAs and that DICER and AGO2 play critical roles in autophagy-mediated regulation of miRNAs. Therefore, we investigated the effect of AMPK activation on autophagy-mediated miRNA processing. Hence, we next tested the miRNA-processing enzyme DICER and the main translation initiation factor AGO2 along with LC3-II (as an indicator of autophagy). BV-2 cells were treated with 2.5 mM metformin, 300 μM AICAR or 50 μM resveratrol for 12 h. Immunoblot results showed that AMPK activators increased the level of LC3-II (Fig. 5b) and subsequently decreased the levels of DICER (Fig. 5c) and AGO2 (Fig. 5d) but did not change the level of Argonaute1 (AGO1) (Fig. 5d).

To further seek evidence for the relationship between AMPK activation and miRNA-30a-5p regulation. BV-2 cells were transfected with Atg5 siRNA for 36 h (Fig. 5k) and then treated with metformin (2.5 mM, 12 h). Immunoblot analysis showed that LC3-II (Fig. 5e, f) was upregulated in metformin-treated cells and that DICER and AGO2 were suppressed (Fig. 5e, g, h). In addition, as shown in Fig. 5i, j, metformin upregulated SOCS3 through the inhibition of miRNA-30a-5p. Atg5 siRNA nearly completely abrogated the effect of metformin. Taken together, these data suggested that AMPK activation upregulated SOCS3 by enhancing autophagy to suppress miRNA-30a-5p.

**Fig. 6:**
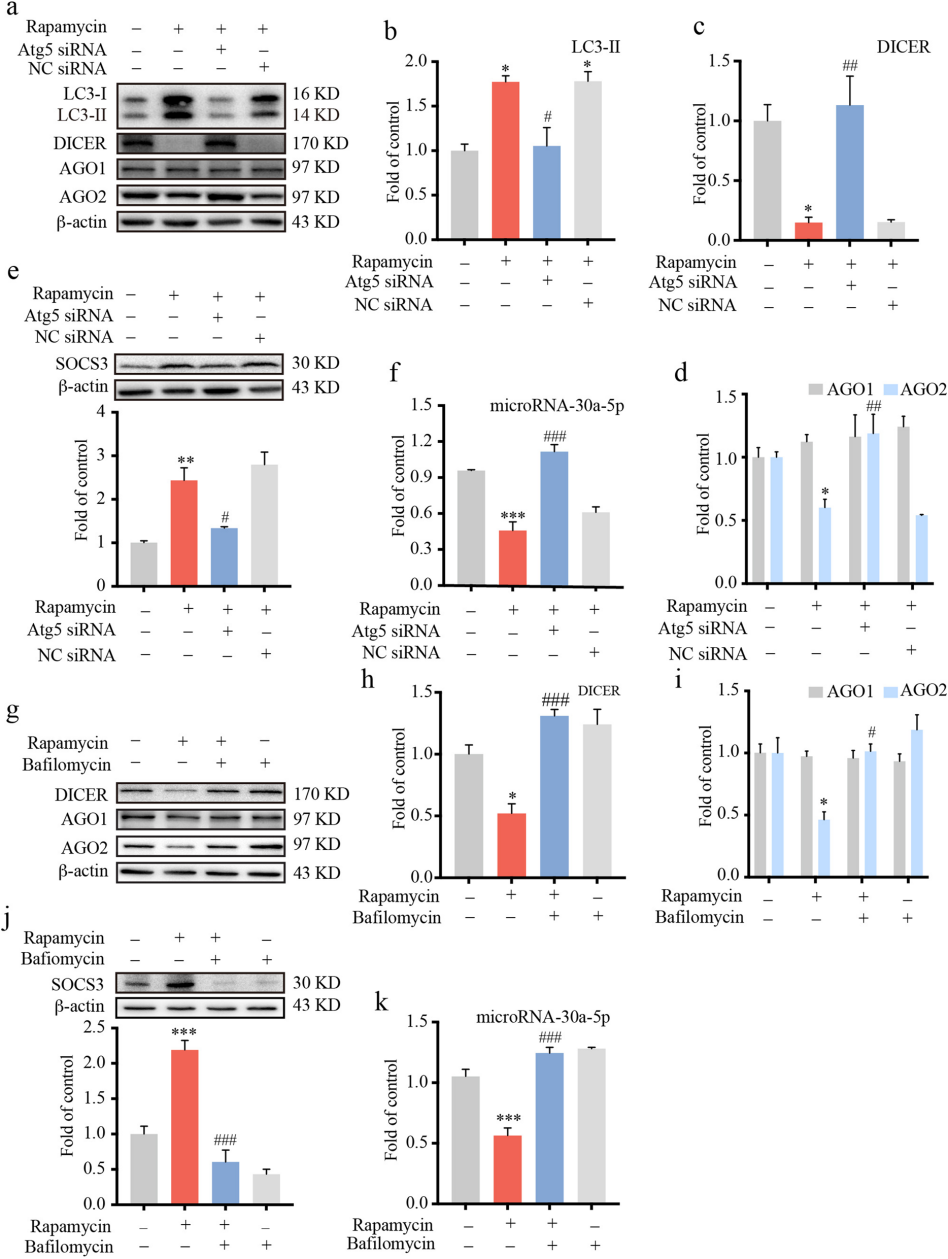
Autophagy inhibited the expression of microRNA-30a-5p by degrading DICER and AGO2. BV-2 cells were transfected with 100 pmol Atg5 siRNA or negative control (NC) siRNA for 36 h and then subjected to rapamycin (200 nM) for 12 h. Representative western blot data show the protein levels of LC3-II, DICER, AGO1, AGO2 and SOCS3 (**a**-**e**) (*n* = 3). **f** The level of miRNA-30a-5p was tested by real-time PCR (*n* = 3). BV-2 cells were treated with bafilomycin (20 nM) for 12 h, followed by rapamycin administration (200 nM) for 12 h. The protein levels of DICER, AGO1, AGO2 and SOCS3 were evaluated by western blot (**g**–**j**) (*n* = 3). **k** The level of miRNA-30a-5p was tested by real-time PCR (*n* = 3). Data were analyzed by one-way ANOVA. *p < .05, **p < .01, ***p < .001 vs. the control group; ^#^*p* < .05, ^##^*p* < .01, ^###^*p* < .001 vs. the rapamycin-treated group

Rapamycin, a special and selective inhibitor of mammalian target of rapamycin (mTOR), can effectively activate autophagy. To investigate the role of autophagy in the regulation of miRNA-30a-5p, BV-2 cells were treated with rapamycin (200 nM, 12 h) with or without Atg5 knockdown. In accordance with previous studies, rapamycin increased the level of LC3-II (Fig. 6a, b) and decreased DICER (Fig. 6a, c) and AGO2 (Fig. 6a, d). This effect of rapamycin was attenuated by Atg5 siRNA. Furthermore, we tested the level of miRNA-30a-5p in BV-2 cells by real-time PCR after rapamycin administration. As shown in Fig. 6f, Atg5 siRNA alleviated the inhibitory effect of rapamycin on miRNA-30a-5p. At the same time, immunoblot results demonstrated that Atg5 siRNA inhibited the effect of rapamycin-induced upregulation of SOCS3 (Fig. 6e). To further confirm the contribution of autophagy to miRNA-30a-5p, we used bafilomycin, an inhibitor of lysosomal acidification, before rapamycin administration in BV-2 cells. As expected, bafilomycin abolished the suppressive effect of rapamycin on DICER (Fig. 6g, h) and AGO2 (Fig. 6g, i). In addition, the data in Fig. 6j, k show that rapamycin increased the expression of SOCS3 via inhibition of miRNA-30a-5p. Bafilomycin abrogated the effect of rapamycin on miRNA-30a-5p and on SOCS3. These data verified that autophagy-mediated inhibition of miRNA-30a-5p is a key step for the upregulation of SOCS3.

### AMPK-autophagy activation suppressed the morphine-induced inflammatory response through the upregulation of SOCS3

**Fig. 7:**
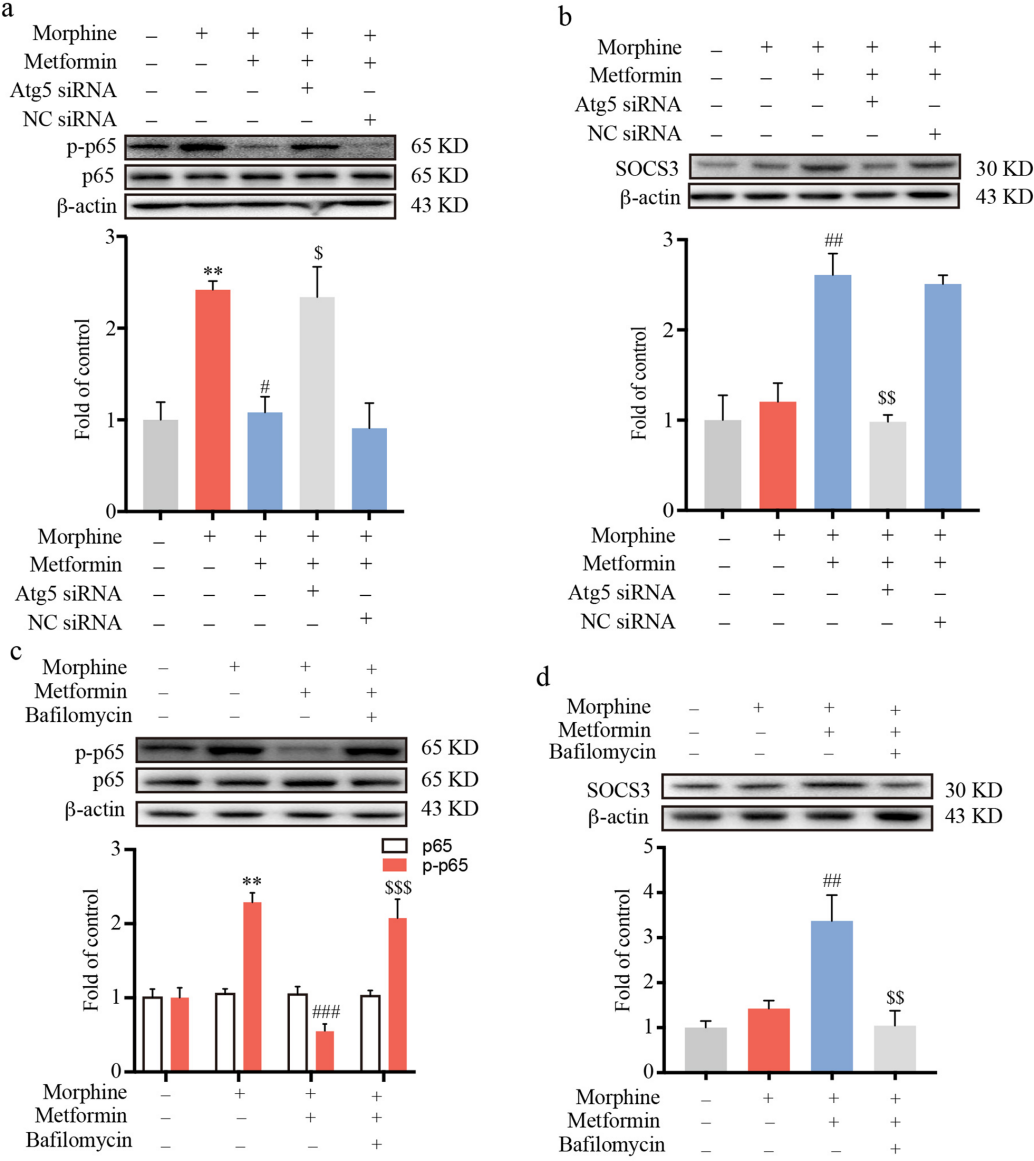
AMPK-autophagy activation suppressed the morphine-induced inflammatory response through the upregulation of SOCS3. **a**, **b** BV-2 cells were transfected with 100 pmol Atg5 siRNA or negative control (NC) siRNA for 36 h and then subjected to metformin (2.5 mM) for 12 h, followed by exposure to morphine (200 µM) for 12 h. The phosphorylation of p65 (**a**) and the protein level of SOCS3 (**b**) were tested by western blot (*n* = 3). **c**, **d** BV-2 cells were pretreated with bafilomycin (20 nM) for 12 h and then subjected to metformin (2.5 mM) for 12 h, followed by exposure to morphine (200 µM) for 12 h. The phosphorylation of p65 (**c**) and the protein level of SOCS3 (**d**) were tested by western blot (*n* = 3). Data were analyzed by one-way ANOVA. **p < .01 vs. the control group; ^#^*p* < .05, ^##^*p* < .01, ^###^*p* < .001 vs. the morphine-treated group. ^$^*p* < .05, ^$$^*p* < .01, ^$$$^*p* < .001 vs. morphine and metformin-coadministered group

To determine whether AMPK-autophagy activation could inhibit the morphine-induced inflammatory response, Atg5 siRNA was utilized to inhibit the autophagy process. BV-2 cells were transfected with 100 pmol Atg5 siRNA or negative control (NC) siRNA for 36 h and then exposed to metformin (2.5 mM, 12 h), followed by morphine administration (200 μM, 12 h). We found that Atg5 knockdown significantly attenuated the anti-inflammatory effect of metformin (Fig. 7a). As expected, Atg5 knockdown also inhibited the upregulation of SOCS3 caused by metformin (Fig. 7b). Furthermore, BV-2 cells were treated with 20 nM bafilomycin for 12 h and then exposed to 2.5 mM metformin for 12 h, followed by morphine stimulation (200 μM, 12 h). Immunoblotting showed that bafilomycin, similar to Atg5 siRNA, alleviated the anti-inflammatory effect of metformin (Fig. 7c, d). Based on the data above, AMPK-autophagy activation suppressed the morphine-induced inflammatory response through the upregulation of SOCS3.

## Discussion

**Fig. 8:**
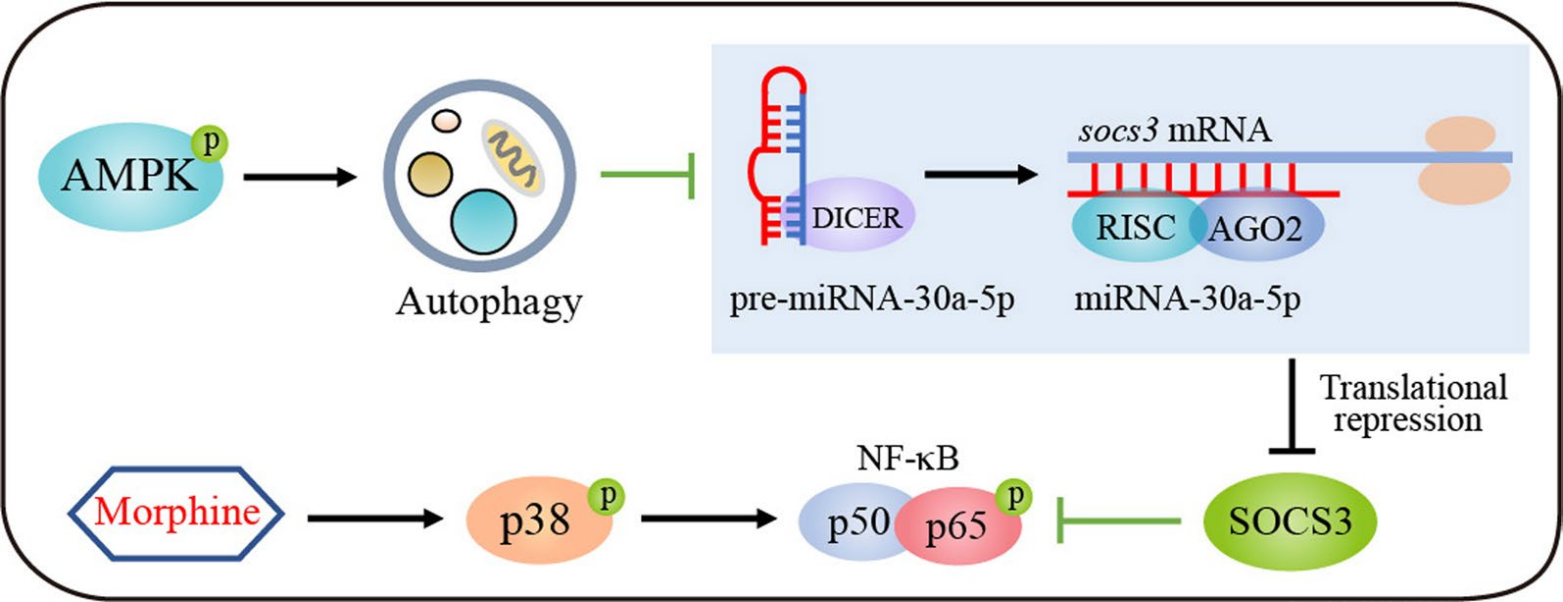
Schematic model indicated that AMPK-autophagy activation upregulated SOCS3 via the inhibition of microRNA-30a-5p by degrading DICER and AGO2. It suppressed morphine-induced neuroinflammation and improved analgesic tolerance

In our present study, miRNA-30a-5p was identified as a key target to improve morphine tolerance. We first demonstrated that a miRNA-30a-5p inhibitor markedly ameliorated morphine tolerance and had an obvious inhibitory effect on morphine-induced activation of microglia and neuroinflammation. Furthermore, activation of the AMPK-autophagy axis inhibited miRNA-30a-5p by degrading DICER and AGO2, leading to the upregulation of SOCS3 in microglia (Fig. 8). Here, we innovatively clarified the role of AMPK-autophagy-mediated anti-neuroinflammatory effects in the treatment of morphine tolerance. Our studies demonstrated that miRNA-30a-5p inhibitor as a nucleic acid reagent could prolong the efficacy of morphine.

It is well-accepted that chronic morphine administration leads to neuroinflammatory responses in the spinal cord, characterized by the release of proinflammatory cytokines [2]. In morphine-evoked neuroinflammation, *N*-methyl-d-aspartate receptor (NMDA-R)-mediated central hyperalgesia plays a vital role in the development of morphine tolerance [26, 27]. It was reported that NMDA-R/nitric oxide (NO) pathway antagonists could enhance the antinociceptive effect of morphine. However, the inhibition of NMDA-R/NO may diminish the anticonvulsant effect of high-dose morphine [28]. In addition, a growing body of reports has indicated that the MAPK family, including p38 MAPK and JNK, is intimately associated with the morphine-induced inflammatory response in microglia. Xiaohui Wang et al. reported that morphine induced neuroinflammation by binding to the TLR4 accessory protein myeloid differentiation protein 2 (MD-2) in a similar way to LPS. Morphine behaved like LPS in the activation of the TLR4 pathway and increased the phosphorylation of p38 [1]. Unfortunately, there are no safe and effective drugs in the clinic for treating morphine tolerance via anti-inflammation. It is urgent to find a new therapeutic target to improve morphine tolerance.

At present, it has been reported that morphine-induced activation of microglia can be prevented by metformin [25], resveratrol [29] or lidocaine [19] via activation of AMPK by targeting the TLR4–MAPK–NF-κB pathway. Given the beneficial effects of AMPK activation, AMPK is heavily pursued as a therapeutic avenue for the treatment of several metabolic diseases, such as diabetes [30], obesity [31] and cancer [32]. In recent years, an increasing number of studies have shown that AMPK activation attenuates pain through anti-inflammatory effects. Evidence has shown that AMPK activation produces an analgesic effect by inhibiting NF-κB activation and reducing the expression of IL-1β in neuropathic pain [33]. Our previous study also illustrated that perisciatic nerve injection of ozone attenuated CCI-induced neuropathic pain through the activation of AMPK. However, the mechanism underlying AMPK activation in inflammatory suppression remains elusive.

Suppressor of cytokine signaling (SOCS) proteins are critical regulators of both innate and adaptive immunity. Increasing evidence is also emerging of the involvement of SOCS proteins in the treatment of neuroinflammation-mediated diseases, such as morphine tolerance [19], chemotherapeutic enteritis [34] and cancer [35]. Among the SOCS family, SOCS3 plays a critical role in suppressing neuroinflammation by negatively regulating the function of TGF-β-activated kinase 1 (TAK1) [15]. Evidence has demonstrated that the effect of SOCS3 on the TLR4 signaling pathway is based on its ability to inhibit the activation of TNF receptor-associated factor 6 (TRAF6) and TAK1, both of which are crucial for TLR4-mediated inflammatory responses. SOCS3 was identified as a key protein at the crossroads of numerous intracellular and pathological events [14]. Based on these studies, agents that increase the level of SOCS3 could potentially be used to treat TLR4-mediated neuroinflammation.

At present, the mechanism underlying the regulation of SOCS3 is massive. Goren et al. reported that the IL-10/STAT3/SOCS3 axis was involved in the anti-inflammatory effect of benznidazole [36]. In addition, rapamycin decreased the levels of TLR-3 and TLR-4 by mediating the TAM-TLR-SOCS3 signaling pathway [37]. Nevertheless, the molecular mechanism underlying SOCS3 regulation is not fully understood. In this study, we found that AMPK activators upregulated the protein level of SOCS3 but did not affect *Socs3* mRNA. This result suggested that posttranscriptional regulation may be involved. MicroRNAs (miRNAs) are endogenous 21–23 nt RNAs that play critical gene-regulatory roles in animals and plants by pairing to the mRNAs of protein-coding genes to direct their posttranscriptional repression. MiRNAs dysregulation causes various diseases [38, 39]. An increasing number of studies have reported that miRNAs are involved in human cancer [40], ischemic stroke [41] and neurodegenerative disorders [42]. However, the significance of miRNA regulation in the treatment of morphine tolerance remains unclear. Based on bioinformatics prediction and experimental verification, we found that miRNA-30a-5p directly targeted SOCS3. We found that AMPK activators specifically decreased the level of miRNA-30a-5p and subsequently increased the level of SOCS3 in microglia (Additional file 1: Fig. S2). Interestingly, AMPK activators did not affect the level of pri-miRNA-30a, which implied an influence on the processing of miRNA-30a-5p.

MiRNAs can be processed by Drosha from pri-miRNAs to precursor format (premiRNAs), which are exported to cytoplasm and then sliced by DICER to miRNA/miRNA* duplex [39]. The duplex is cleaved into mature miRNA from one strand, and the other strand miRNA* is degraded. The muture miRNA is loaded with AGO2 into the RNA-induced silencing complex (RISC) to silence target mRNA [43]. Autophagy, a degradative process in which cytoplasmic material is targeted into double-membrane vacuoles, is recognized to critically contribute to cellular homeostasis. It was reported that autophagy regulated miRNA activity through the degradation of DICER and AGO2 [20]. Our data showed that the downregulation of miRNA-30a-5p was mediated by AMPK activators depending on autophagy activation. Rapamycin, an inhibitor of mammalian target of rapamycin (mTOR), could decrease miRNA-30a-5p. Pharmacological or genetic inhibition of autophagy dramatically attenuated the effect of rapamycin on miRNA-30a-5p. This result suggested that rapamycin triggered autophagy to regulate miRNA-30a-5p processing. Xu et al. reported that mTOR was activated in the rat spinal dorsal horn after repeated morphine administration. Activated mTOR increased the expression of the key tolerance-associated protein neuronal NOS (nNOS). Suppression of mTOR with rapamycin significantly improved morphine tolerance [24]. Notably, our study illustrated a new mechanism underlying mTOR inhibition in the treatment of morphine tolerance. It depended on the induction of autophagy.

We know that morphine is mostly used for treating severe pain, especially cancer pain. Cancers can upregulate autophagy to survive and increase growth and aggressiveness. Autophagy promotes their growth, survival, invasion and metastasis [44]. It plays a tumor-suppressive or tumor-promoting role in cancer progression. In early tumorigenesis, autophagy prevents tumor initiation and suppresses cancer development. Once the tumor progresses to the late stage, autophagy contributes to the survival and growth of eatablished tumors and promotes the aggressiveness of cancer [45, 46]. Accordingly, inducing autophagy may not be the best way to attenuate morphine tolerance by upregulating SOCS3 through posttranscriptional regulation. To effectively relieve pain and not influence cancer treatment, we utilized synthesized miRNA-30a-5p inhibitor to directly suppress miRNA-30a-5p and subsequently increase the level of SOCS3. Our data showed that a miRNA-30a-5p inhibitor could inhibit neuroinflammation and largely improve morphine tolerance. Chemically modified nucleic acid drugs are currently used and have become an effective treatment strategy. Oligonucleotides can be used to regulate gene expression by pairing with DNA, mRNA or noncoding RNA. At present, several oligonucleotide drugs are approved by the FDA for therapy [47]. Accordingly, miRNA-30a-5p inhibitor may bring bright prospects for the treatment of morphine tolerance. It also signifies that epigenetic therapies may be the future direction of morphine tolerance treatment.

## Conclusions

In summary, our study revealed that miRNA-30a-5p could be a key target in the treatment of morphine tolerance. We demonstrated that miRNA-30a-5p inhibitor as a nucleic acid reagent could prolong the efficacy of morphine. Activation of the AMPK-autophagy axis could inhibit miRNA-30a-5p by degrading DICER and AGO2 and ameliorate morphine tolerance by SOCS3-mediated anti-inflammatory effect.

## Supplementary Information

**Additional file 1: Figure S1.** Metformin did not affect the expression of SOCS3 in either SH-SY5Y cells or C8-DA cells. (a) SH-SY5Y cells were subjected to metformin (2.5 mM) for 12 h. (b) c8-DA cells were treated with metformin (2.5 mM) for 12 h. The protein level of SOCS3 was tested by western blot (*n* = 3). Data were analyzed by Student’s *t*-test. **Figure S2.** Metformin did not affect the level of miRNA-30a-5p in either SH-SY5Y cells or C8-DA cells. (a) SH-SY5Y cells were treated with metformin (2.5 mM) for 12 h. (b) c8-DA cells were treated with metformin (2.5 mM) for 12 h. The level of miRNA-30a-5p was calculated by real time PCR (*n* = 3). Data were analyzed by Student’s *t*-test.

## Data Availability

All data generated and analyzed during this study are included in this published article and its additional information.

## Abbreviations

AMPK: Adenosine 5′-monophosphate-activated protein kinase
AGO1: Argonaute1
AGO2: Argonaute 2
Atg5: Autophagy related protein 5
DAPI: 4,6-Diamino-2-phenylindole
GFAP: Glial fibrillary acidic protein
i.t.: Intrathecal
Iba1: Ionized calcium-binding adapter molecule 1
IL-1β: Interleukin-1β
MAPK: Mitogen-activated protein kinase
MPE: Maximal possible effect
mTOR: Mammalian target of rapamycin
NeuN: Neuronal nuclear protein
NF-κB: Nuclear factor-κB
NMDA-R: *N*-Methyl-d-aspartate receptor
NO: Nitric oxide
PBS: Phosphate-buffered saline
RIPA: Radioimmunoprecipitation assay
siRNA: Small interfering RNA
SOCS3: Suppressor of cytokine signaling 3
TAK1: TGF-β-activated kinase 1
TLR4: Toll-like receptor 4
TNF-α: Tumor necrosis factor-α
TRAF6: TNF receptor-associated factor 6
