# Supplementary material for: AMPK-autophagy-mediated inhibition of microRNA-30a-5p alleviates morphine tolerance via SOCS3-dependent neuroinflammation suppression
Source: J Neuroinflammation. 2022 Jan 29;19:25. doi: 10.1186/s12974-022-02384-3 (PMC8800317; doi:10.1186/s12974-022-02384-3)
Supplement: Supplementary file 1 — Additional file 1: Figure S1. Metformin did not affect the expression of SOCS3 in either SH-SY5Y cells or C8-DA cells. (a) SH-SY5Y cells were subjected to metformin (2.5 mM) for 12 h. (b) c8-DA cells were treated with metformin (2.5 mM) for 12 h. The protein level of SOCS3 was tested by western blot (n = 3). Data were analyzed by Student’s t-test. Figure S2. Metformin did not affect the level of miRNA-30a-5p in either SH-SY5Y cells or C8-DA cells. (a) SH-SY5Y cells were treated with metformin (2.5 mM) for 12 h. (b) c8-DA cells were treated with metformin (2.5 mM) for 12 h. The level of miRNA-30a-5p was calculated by real time PCR (n = 3). Data were analyzed by Student’s t-test. [file 12974_2022_2384_MOESM1_ESM.docx]

**
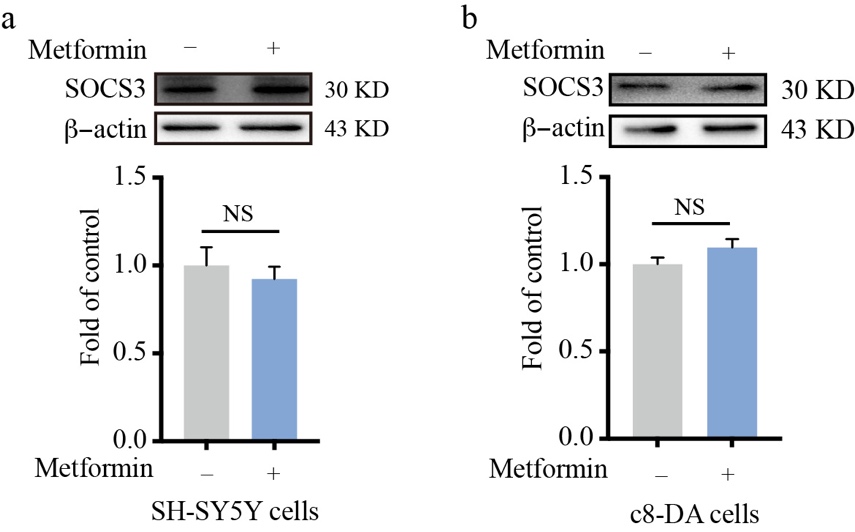
**

**Fig. S1.** (**a**) SH-SY5Y cells were subjected to metformin (2.5 mM) for 12 h. (**b**) c8-DA cells were treated with metformin (2.5 mM) for 12 h. The protein level of SOCS3 was tested by western blot (n = 3). Data were analyzed by Student’s *t*-test.


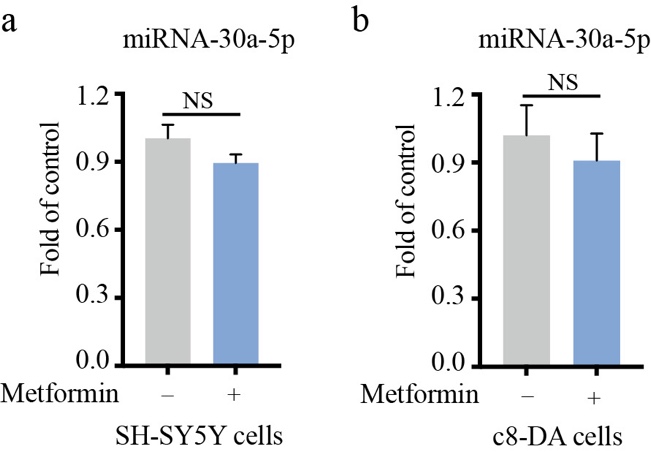


**Fig. S2.** (**a**) SH-SY5Y cells were treated with metformin (2.5 mM) for 12 h. (**b**) c8-DA cells were treated with metformin (2.5 mM) for 12 h. The level of miRNA-30a-5p was calculated by real time PCR (n=3). Data were analyzed by Student’s *t*-test.
